# Lessons Learned in Promoting Evidence-Based Public Health: Perspectives from Managers in State Public Health Departments

**DOI:** 10.1007/s10900-018-0494-0

**Published:** 2018-03-02

**Authors:** Peg Allen, Rebekah R. Jacob, Meenakshi Lakshman, Leslie A. Best, Kathryn Bass, Ross C. Brownson

**Affiliations:** 1 Prevention Research Center, Brown School, Washington University in St. Louis, One Brookings Drive, Campus Box 1196, St. Louis, MO 63130 USA; 2 Pennsylvania Department of Health, and Senior Consultant, National Association of Chronic Disease Directors, Orrtanna, PA USA; 3 Division of Public Health Sciences and Alvin J. Siteman Cancer Center, Washington University School of Medicine, Washington University in St. Louis, St. Louis, MO USA

**Keywords:** Evidence-based public health, Public health departments, Administration, Chronic disease prevention, Health promotion

## Abstract

Evidence-based public health (EBPH) practice, also called evidence-informed public health, can improve population health and reduce disease burden in populations. Organizational structures and processes can facilitate capacity-building for EBPH in public health agencies. This study involved 51 structured interviews with leaders and program managers in 12 state health department chronic disease prevention units to identify factors that facilitate the implementation of EBPH. Verbatim transcripts of the de-identified interviews were consensus coded in NVIVO qualitative software. Content analyses of coded texts were used to identify themes and illustrative quotes. Facilitator themes included leadership support within the chronic disease prevention unit and division, unit processes to enhance information sharing across program areas and recruitment and retention of qualified personnel, training and technical assistance to build skills, and the ability to provide support to external partners. Chronic disease prevention leaders’ role modeling of EBPH processes and expectations for staff to justify proposed plans and approaches were key aspects of leadership support. Leaders protected staff time in order to identify and digest evidence to address the common barrier of lack of time for EBPH. Funding uncertainties or budget cuts, lack of political will for EBPH, and staff turnover remained challenges. In conclusion, leadership support is a key facilitator of EBPH capacity building and practice. Section and division leaders in public health agencies with authority and skills can institute management practices to help staff learn and apply EBPH processes and spread EBPH with partners.

## Background

Evidence-based public health (EBPH) practice involves complex processes to improve population health and reduce disease burden [1–3]. Examples of these processes include the systematic use of population risk factor and disease burden data, intervention evidence, and community assessments to make programmatic decisions and set priorities; program planning frameworks; participatory decision-making; program evaluation; use of what is learned to improve implementation; and sharing what is learned [1, 4, 5]. In the U.S., multiple national agencies push for EBPH. EBPH is embedded in the Public Health Foundation’s ten essential public health services [6] and EBPH training and documentation are required for state and local public health department accreditation by the Public Health Accreditation Board [7]. The Community Guide and other entities publish evidence recommendations from systematic reviews of interventions [8].

The Centers for Disease Control and Prevention (CDC) and other federal agencies increasingly require the use of evidence-based approaches when funding population-based chronic disease prevention and control units in state public health departments to fund and support local implementation. In chronic disease prevention, many of the evidence-based interventions promoted by The Community Guide and CDC, such as those to reduce tobacco use or increase physical activity opportunities, are multilevel and involve complex system-wide and/or environmental and policy changes [8]. Such changes involve collaboration with organizations in sectors within and outside of health [8, 9].

Organizational structures and processes can facilitate capacity building for EBPH. Leadership commitment to EBPH is consistently described as a key facilitator in literature reviews from public health, healthcare, business, and qualitative studies with local health departments in Canada and state injury prevention agencies in Australia [2, 10–14]. Additional components include ongoing workforce training for EBPH, a supportive organizational climate and culture in which EBPH is the accepted norm, relationships and partnerships with aligned missions and participatory decision-making, and transparent financial practices such as clear expectations and processes for EBPH components in requests for proposals and contracts [2, 11]. Barriers to EBPH have been identified in earlier studies [15, 16], but little is known about U.S. state public health department manager views on facilitators of EBPH capacity building [17].

The purpose of this multi-state qualitative study was to identify facilitators of EBPH capacity building in state health department chronic disease prevention and management units.

## Methods

### Study Design

**Fig. 1:**
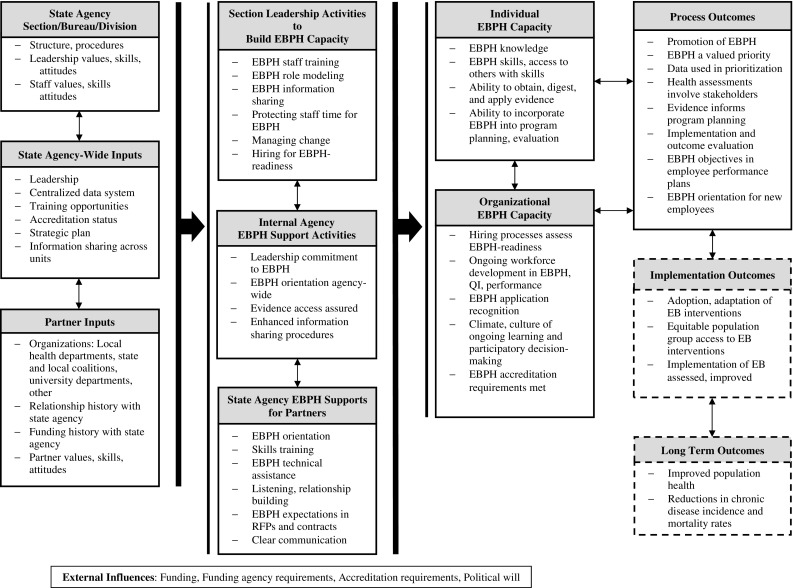
Map of state health department evidence-based public health capacity building

Interviews were conducted in 2016 as part of a larger multi-year project. The purpose of the larger study was to better understand how university-based applied researchers could support state health departments to enhance capacity for evidence-based chronic disease prevention. The multi-year study was a group randomized trial with state health department chronic disease units (states) randomly selected and assigned to receive study team EBPH training and follow-up support (six states) or receive links to electronic resources for EBPH and participate in data collection (six states), with opportunity to receive study team training after data collection. Methods and pre-post survey findings from the larger study are reported elsewhere [18–20]. While the larger study sought to examine effects of study participation between the control and intervention sites [18], this qualitative study sought to explore perspectives on facilitators and barriers to EBPH generally across the sample of 12 state health departments. Figure 1 shows a map of state health department EBPH capacity building informed by a literature review, the work of Kramer and Cole, and study team findings [2, 11, 21, 22]. The study obtained human subjects exempt approval from the Washington University Institutional Review Board.

### Interview Participants and Data Collection

A purposive sample of interview participants was selected by the chronic disease directors in the 12 states in collaboration with the study team. Interview participants were mostly chronic disease unit (section, bureau, division) leaders and program managers. The respondents were often three to five layers below the state health officer. A few managers working cross-agency in quality improvement or performance management were also interviewed. After interview participants were invited by email and provided informed consent, a one-hour interview appointment was set for a later date, allowing further opportunity to decline at any time. Interviews were conducted in 2016 by phone by the study team (LB, PA) and ranged from 45 to 60 min in length. LB interviewed individuals with whom PA had worked during the study; PA interviewed others.

### Measures

The structured interview guide contained open-ended questions on activities of chronic disease units to support EBPH capacity building and application, internal and external influences, facilitators, challenges, and recommendations for the future. The first question was “how does your work unit use management practices to support evidence-based processes”. Participants were also asked to describe “the work unit environment as it relates to using evidence-based processes”, “any additional evidence-based process supports at the division or agency level”, acceptance of practices intended to support EBPH, what facilitated getting supportive practices in place, challenges encountered and how challenges were addressed, and “supports and expectations created for external partnering organizations for evidence-based processes”.

### Data Analyses

Each phone interview was audio-recorded with participant permission and transcribed verbatim. Each de-identified transcript was independently coded in NVIVO 10 qualitative software by two study team members who then met to reach consensus on any disagreements in text blocks and codes [23]. Queried texts were then exported from NVIVO into Word for review and mark-up. Interim matrices and tables were created to summarize findings. Coded texts and interim tables were reviewed to identify themes and illustrative quotations [24, 25].

## Results

Fifty-one of 54 invited state health department managers from 12 states completed 45–60-min phone interviews in 2016. Most (74%) participants were women, 31% held a public health graduate degree, and 37% had a master’s or doctorate degree in another field. Participants had worked in their positions an average of 5.9 years, in their agency 12.7 years, and in public health for 20.1 years. Participants directly supervised an average of 8.3 employees. At the time of the interviews, four of the 12 state health departments were accredited by the Public Health Accreditation Board, four were actively preparing to apply, two had temporarily abandoned preparations due to competing priorities and/or changes in leadership, and two had recently started preparations.

In addition to the “constant drum beat” of federal funding and national accreditation requirements for evidence-based approaches, four themes emerged as key facilitators to building capacity for integrating EBPH into day-to-day practice: leadership support; incorporation of EBPH into agency structures and processes; commitment to professional development and hiring of staff with training and/or experience in EBPH; and external partner relationship building, training, and technical assistance. In addition to budget cuts and funding uncertainty, main challenges discussed by participants were lack of time; lack of external political will or support for EBPH; and staff turnover. Interview findings mapped onto the EBPH capacity building framework shown in Fig. 1.

### Leadership Support

Most participants discussed section and division leadership support as key levels of support that furthered EBPH. In most states, section and division leaders had to advocate for EBPH both up and down the agency hierarchies. In the two states in which participants described their agency-wide leaders as active drivers of EBPH, participants viewed such top-down support and requirements as crucial. In the other states, top agency leader nods of encouragement or permission to proceed were seen as beneficial but not essential to moving forward within sections or divisions. Section leaders were viewed as the central EBPH role models, but some program managers or section leadership team members also served as EBPH champions.

“Leadership modeling” involved far more than just valuing EBPH and encouraging it. Section or division leadership support meant being a “key driver”, “role model”, “champion” of EBPH, and having “the vision to see it move forward”. “Staff seeing that she was excited about this was really, really helpful…seeing her promote this has been really great.” Another participant noted the section leader “has given us energy that we need to feel comfortable and competent.” “Ability to speak to the evidence, and talk about evidence evaluation, and changing programs according to what you find in evaluation, it helped us as we wrote a new DOH strategic plan.” “To have a leader who was sold on it [EBPH] and was continuing to bring it up and lead by example, was an important contributor to why it got incorporated more.”

The main way leaders showed their support of EBPH was creating “continued conversation” and regularly asking for evidence in meetings. Leaders would ask “Well, is that evidence-based? What’s the science behind that idea or that proposed objective?” “She wants to know, do we know that this is something that will work, or can work, and what’s going to back that up? So are we making decisions based on the best evidence that we have?” “How do you know that you’re making a difference? What data sources do you have that could track your success? And how do you know you’re reaching the people you intend to reach?” “Part of the way he shows his support is by communicating about it and also by making specific requests when we present potential decisions…and so he has something to present to his constituents and his leadership”.

Leaders provided “space” for EBPH and a supportive organizational environment. Leaders gave program managers and staff “permission”, time, and “room” to find and think through the evidence, “and to use that information as we are developing programs, developing policies.” Leaders ensured that “we allocate resources efficiently, responsibly, transparently, and we know that’s through using evidence-based strategies and programs, we don’t like to veer off that pathway”. EBPH is “ingrained”, “understood”, “part of the culture”, “inherent in how we work on program efforts”, “the way we do business”.

“We use evidence-based practices because that’s what good public health is.”

“Climate has changed to the point where people [staff] actually get upset if we aren’t doing something that’s evidence-based.”

### Agency Structures, Culture and Processes

In addition to leadership support, managers emphasized the importance of developing documented systems, structures, and procedures to ensure EBPH incorporation became permanent. “There’s a lot of culture here around documenting processes and testing to see if they can be improved.” At least two state health departments restructured their formal administrative units to better share information and support EBPH across program areas. All 12 participating state health departments instituted regular meetings across chronic disease prevention and/or broader units to provide EBPH messaging and share information, plans, and successes. More than half of the states capitalized on agency-wide development or expansion of centralized data systems to increase internal and partner access to data and intervention evidence and share performance measures. Half of the chronic disease units incorporated EBPH learning and practice objectives into employee evaluations within their states’ existing performance review systems. Managers described increased commitment to hire evaluators and epidemiologists to support EBPH, and to hire program staff trained and/or experienced in EBPH. Several managers formalized this commitment by including expectations for EBPH in job descriptions and interview questions. Each chronic disease unit transitioned to requiring EBPH in contracts that funded in-state partners.

“If you don’t change the systems and structures down to the level we were talking about, about changing the questions that are asked on a proposal…then the change is dependent on that person [leader] being there and continuing to be inspiring.”

### Workforce Development

Leaders realized that ongoing periodic training was also needed to ensure staff members were well prepared for EBPH. All participating states provided or hosted brief EBPH-related overview and topical skills trainings for their chronic disease prevention staff to some extent, as well as quality improvement and/or performance management training for staff agency-wide. “Adequate training in evidence-based practice is most useful, without that we can’t really move forward at state or local level.” Half of the states initiated new employee orientation to EBPH. Managers acknowledged state health department accreditation efforts had provided impetus and agency support for EBPH workforce development and improved documentation. “So we are all much more in tune to documenting what we do and making sure we understand why.”

### External Partner Relationships

The managers emphasized the importance of building and maintaining relationships with external partners while promoting EBPH practice through contracts. Managers said they and their state-level staff needed “the ability to develop strong, healthy, productive relationships with the people around the state that are doing the work.” Interview participants stressed the importance of compromise, listening, getting input, building trust, and being transparent about decision-making processes. They also spoke about avoiding being directive or pushing too hard or fast, instead “needing to be thoughtful and realistic, it brings a larger majority along together”. It was important “to listen to how things seem to be going according to our partners’ perspectives, what they see as barriers or challenges, and then to be responsive to that.” “Once you get their trust and they’re [partners] onboard, they understand that what’s evidence-based is going to make the biggest difference in their communities.”

“Sometimes partners are very passionate and advocate for their particular issue that they want addressed that may not necessarily be what the evidence is showing as the biggest issue…You have to balance, think about the relationships, and be able to address the relationships.”

“And so what we work to do is to be really clear on how we’re establishing priorities and what kinds of criteria we’re considering. And taking input and then reaching a compromise and letting people know that we value their input and we take it very seriously and take it to heart.”

In addition to relationship building and maintenance, participants discussed the importance of EBPH training and technical assistance they provided to external partners. States provided multiple brief webinars, in-person partner trainings on program evaluation, grant writing, or other topics, and emailed notices of trainings provided by others. EBPH technical assistance was through one-on-one or small group phone calls and in-person visits. Technical assistance included program planning, discussion of contract menu options, guidance on program evaluation, orientation of new partners, review of “what went well, what didn’t go as well as hoped” and “open, honest dialogue with folks about how can we help you make this work in your community.” State health department staff then wrote EBPH objectives and menus of evidence-based strategies, which were incorporated into requests for in-state proposals and contracts.

To sustain EBPH capacity and practice, managers emphasized the importance of documented agency structures and processes to make supports for EBPH practice permanent, documentation and use of common language for EBPH internally across program areas and with external partners, and ongoing refresher trainings. Managers also spoke about the importance of “continuing the conversation” about EBPH through “constant reinforcement of what is considered EBPH”, cross-program meetings to share EBPH examples and evaluation plans across program areas and provide EBPH reinforcement reminders, starting program planning with the evidence and data, and incorporating EBPH into employee performance evaluations.

### Challenges

Four main challenges to EBPH emerged: (1) funding uncertainties or budget cuts, (2) lack of time, (3) lack of political will or support for EBPH, and (4) staff turnover. Participants attributed the time challenge to inadequate staffing levels and the extra time EBPH planning took. Staff turnover was a common frustration. “You get staff trained and they leave, so you lose that piece that was gained.” Managers viewed EBPH orientation of new staff and periodic brief trainings as partial solutions. Offering flex scheduling, learning opportunities, and resources needed for the work were strategies managers were using to hire and retain qualified staff despite non-competitive low pay. While managers in three state health departments said they enjoyed political support for EBPH, participants in the other states expressed frustration at sometimes being asked by agency or state political leaders to take approaches not grounded in evidence. Managers said competing priorities or “hot issues” sometimes put evidence-based chronic disease prevention aside. Lack of evidence in some program areas or with some population groups was also mentioned.

### Participant Recommendations

Participant recommendations to further incorporate and sustain EBPH practice largely mirrored the findings covered above. Participants in 10 states recommended reinforcing an organizational climate supportive of EBPH. Managers stated this could be accomplished through establishment of EBPH as a foundational or guiding principle and increased incorporation of EBPH in goals and work plans, commitment to EBPH through formal EBPH accountability procedures, frequency of employee and contractor monitoring for EBPH application, communication about EBPH within the agency, and continued incorporation of EBPH in strategic planning. Participants recommended continuing education including ongoing EBPH orientation for new staff and training in program evaluation, leadership development training and mentoring, and training on how organizations change, benefits of organizational change, and staff roles and coping strategies in organizational change. “I think in order to keep it [EBPH] building and growing as you bring on new staff it has to be a part of orientation.” Participants recommended training sessions be recorded for later review, possibly in an interactive webinar format.

## Discussion

To build EBPH capacity and supports, chronic disease directors and program managers in 12 state health departments emphasized the importance of section and division leadership support, creation of in-house procedures to make EBPH processes more permanent and less dependent on individual EBPH champions, ongoing workforce development in EBPH, and creation of EBPH expectations and capacity-building supports with external partners.

Leadership support provided by chronic disease directors and division leadership to build EBPH capacity and practice included role modeling, consistently asking what the evidence was for each planned or proposed approach, creating a supportive work environment for EBPH, and giving staff time to obtain and digest evidence. Leadership support is considered a key component in other studies as well [10, 12–15, 26–28]. Aarons encourages leaders to role model and coach staff on EBPH [10]. Studies with a public health department in Canada emphasize the support of senior influential leaders that can create a multi-year vision for EBPH, role model development of new knowledge and skills in EBPH, and dedicate staff time for review of evidence [13, 14]. Leadership who encourage the dedicated use of staff time to identify and define evidence and data to support EBPH in policy and program planning can offset the commonly cited barrier of lack of time for EBPH [13–15, 27]. In addition to allocating staff time, chronic disease directors can facilitate supportive organizational climates and cultures for EBPH by committing to EBPH capacity building as a process that takes multiple years, committing to EBPH practice for the long-term, communicating expectations, giving staff time to get comfortable with new procedures and processes, praising staff EBPH skill acquisition and application, listening to staff suggestions, incorporating staff in decision-making, recognizing staff, and communicating successes [2, 11, 12, 14, 27, 29]. Public health entities increasingly provide leadership development opportunities and training sessions on change management for states.

Leaders at several levels in public health agencies need leadership and management skill development opportunities and support [30]. State agency managers came to public health from a variety of fields. Most rose through the agency hierarchies without formal education in leadership and management. To address this need, public health leadership and change management trainings are increasingly available through entities such as Regional Public Health Training Centers, the National Network of Public Health Institutes, the Public Health Foundation, CDC, and universities. Professional groups such as the National Association of Chronic Disease Directors provide a variety of activities to build leadership and capacity (e.g., Chronic Disease Academies, Peer Learning Networks) (http://www.chronicdisease.org/). Section, bureau, and division managers also need flexibility to institute procedural changes such as those managers in this study described and to create environments supportive of EBPH [10], which would be further enhanced through support for attendance at change management trainings from key agency leadership.

Procedural changes within public health agencies can help ensure application of EBPH processes, as discussed here and found in the literature. Participants in the present study cited leader and staff turnover, which is a documented problem [31, 32]. To be less dependent on individual champions, participants emphasized incorporation of evidence in internal planning processes and EBPH language and expectations in requests for proposals and contracts. Establishment of regular meetings and communication to share EBPH processes across program areas was another procedure used in the current study and elsewhere [13, 33, 34], as well as use of internal centralized data and performance management systems to gather information for decision-making [12].

Workforce development for EBPH requires a long-term commitment and multi-faceted approaches [11, 35]. In a local public health department in Canada, this commitment included reallocation of vacancies for workforce development positions [13, 14]. As participants pointed out, EBPH orientation is needed for new employees. Training in facets of EBPH such as program evaluation and communicating evidence with decision-makers can improve individual skills [36]. Hiring staff with experience or training in EBPH and incorporation of EBPH into employee performance objectives and feedback are also important aspects of workforce development for EBPH [2].

Provision of training and technical assistance with external partners is also essential to advance the work of state health department chronic disease units, since local partners both within and outside of the public health sector implement complex multi-level evidence-based approaches [2, 9, 11, 37]. One of the ten essential public health services is to mobilize community partnerships to address public health issues [6, 9]. In population-based chronic disease prevention and management, the typical flow of funds is from federal agencies to state health departments, who in turn fund local partners. While some partners provide EBPH training and expertise, others need skill-building opportunities in community health assessment, managing competing priorities, adapting evidence-based approaches for specific setting and population groups, and communicating evidence with policy makers. In a U.S. study of cancer control coalition partners, Steele et al. found partners especially noted challenges in adapting and evaluating implementation of evidence-based approaches [37]. Local partners in a community health improvement initiative in England found top-down or rigid communication styles and inadequate sharing of research evidence and practical experience hampered the partnership work and initiative [38]. Alignment of partner organization missions and opportunities for partners to learn alongside each other and with governmental public health agency staff can facilitate partnerships to improve population health [2, 11].

In conclusion, leadership support at the section, bureau, and division levels is a key facilitator of EBPH capacity building and practice. As discussed by study participants, even if political or agency-wide leadership is lacking, section and division leaders with authority and skills can institute management practices to help staff learn and apply EBPH processes and spread EBPH to partners. Section and division leaders need training and support to do this. In addition to leadership support, individual and organizational EBPH capacity can be enhanced through ongoing training and technical assistance with staff and partners, use of information systems that cross program areas coupled with clear and transparent expectations for EBPH practice in internal documents and external requests for proposals and contracts.
